# Relations Between Parental Emotion Talk and Preschoolers’ Emotion Expressions in Low-Income Chinese American and Mexican American Families

**DOI:** 10.3390/children12010052

**Published:** 2025-01-02

**Authors:** Megan Chan, Michelle Taw, Nancy Eisenberg, Qing Zhou

**Affiliations:** 1 Department of Psychology, University of California Berkeley, Berkeley, CA 94720, USA; 2 Department of Psychology, Arizona State University, Tempe, AZ 85281, USA; nancy.eisenberg@asu.edu

**Keywords:** parental emotion talk, preschoolers’ emotion expressions, culture

## Abstract

**Background/Objectives**: Preschool children learn to express emotions in accordance with sociocultural norms. Parental emotion talk (ET) has been theorized to shape these processes. Limited research has examined preschoolers’ observed emotion expressions and emotion-related behaviors in culturally diverse samples. We sought to explore the following: (1) cultural group differences and similarities in observed emotion expressions (anger, sadness, and positive emotions) and emotion-related behaviors between Chinese American and Mexican American preschoolers, and (2) the concurrent links between parental ET and children’s emotion expressions. **Methods**: In a sample of 86 children (age range = 38 to 70 months, 62% girls) from low-income immigrant families (Mexican Americans/MA = 43 and Chinese Americans/CA = 43), the observed children’s emotion expressions and emotion-related behaviors were coded based on a frustration-eliciting task. Parental ET quality and quantity were coded from transcripts of a parent–child shared reading task. **Results**: MA children expressed more anger and sadness, but the two groups did not differ on positive emotions or emotion-related behaviors. Multiple regressions showed that children whose parents engaged in more ET expressed higher levels of anger and sadness and used more non-feeling state languages than children whose parents engaged in less ET. **Conclusions**: The results revealed cultural variations in preschool-age children’s emotion expressions and provided support for associations between parental ET and children’s emotion expressions.

## 1. Introduction

Parental emotion talk (ET), a common emotion socialization practice during early childhood, plays a critical role in shaping children’s socioemotional competence [1]. Despite the acknowledgement that how parents socialize children’s emotions depend on sociocultural norms and values [1], there is a dearth of research on parental ET in non-Western families and its links to children’s emotion expressions. As the number of immigrant families continues to rise in the United States and elsewhere, it is crucial to understand parental use of emotion talk and its roles in children’s socioemotional development in immigrant families. Such knowledge can inform the development and dissemination of culturally competent parent education and family engagement programs in culturally diverse and immigrant communities. To fill these gaps, the present study sought to explore the following (1) cultural group differences and similarities in emotion expressions (anger, sadness, and positive emotions) and emotion-related behaviors (attention to target, gaze aversion, and verbalization) between Chinese American and Mexican American children using an observational approach, and (2) the concurrent links between parental ET and children’s emotion expressions.

### 1.1. Preschool-Age Children’s Emotion-Related Behaviors and Emotion Expressions

The ability to express emotions in a regulated and appropriate manner is a crucial component of emotional competence in Western cultures [2]. With the development of cognitive, language, and motor skills, preschool-age children learn to regulate their emotions and adapt emotion expressions in alignment with social and cultural norms and expectations [2]. Researchers have identified several types of emotion-related behaviors in young children during emotion-evoking situations. For example, attention control refers to the ability to focus or shift one’s attention voluntarily to facilitate emotion management [3]. Gaze aversion (averting one’s gaze or looking away) is a basic form of self-distraction or attention control [4]. As children develop more sophisticated verbal skills, they increasingly utilize language as a tool for regulating emotions [5]. As such, self-verbalization (including labeling emotions) is commonly observed among preschoolers in emotion-evoking situations [6]. In sum, preschoolers’ emotion-related behaviors reflect their developing emotion regulation skills.

Children’s emotion-related behaviors often co-occur with and are thus related to their expressions of positive and negative emotions, although the direction of such associations varies by valence and situational contexts. For example, in predominantly White samples, positive relations were observed between preschoolers’ expressions of positive emotions (e.g., smiling) and fidgeting [7,8]. Using behavioral data from the same sample of preschoolers as the present paper but assessed with a different paradigm (unfair sharing), we found that children’s gaze aversion was positively associated with negative emotion expressions, whereas fidgeting was positively associated with positive emotion expressions and negatively associated with negative emotion expressions [9]. Because the relations between emotion expressions and emotion-related behaviors vary by situations [4], it is important to investigate these associations in different situations to get a better understanding of young children’s emotion regulatory processes.

### 1.2. Parental Emotion Talk and Its Links to Children’s Emotion Expressions

Emotion talk (ET) refers to conversations involving the labeling of emotions, references to the cause or elicitors of emotions, and the appropriateness or consequences of emotions and emotion-related behaviors [10]. Parental ET is theorized to shape children’s emotion expressions through multiple pathways (e.g., emotion arousal, emotion understanding, and emotion control values). First, by engaging in ET, parents teach, coach, and model constructive emotion regulatory strategies (e.g., labeling, validating, and cognitive restructuring) and culturally appropriate emotion expressions. Second, parental ET or other mental state talk can promote children’s emotion understanding, theory of mind, perspective taking, and emotion vocabulary [11], which in turn facilitate emotion regulation and expression [12]. Third, parents’ use of emotion language and modeling of emotion regulation strategies can shape children’s emotion control values [13]. Research conducted with adults showed that individuals’ emotion control values (e.g., the beliefs that one should or can control one’s emotions) are associated with their emotion regulation strategies and emotion-related behavioral and physiological responses [14,15].

Consistent with these theories, the quality and elaborateness of parental ET has been linked to children’s higher effortful control (a temperament-based self-regulatory process related to emotion regulation), better emotion understanding, and more prosocial behaviors. For example, a latent factor reflecting both the quantity and elaborateness of parental ET predicted school-age children’s higher effortful control in a longitudinal study of Chinese American immigrant families [16]. In a sample of upper to middle-class, predominantly White families, the mothers’ emotion explanations were associated with preschoolers’ greater emotion situation knowledge and greater prosocial behaviors [17].

It is important to note that the relation between parental ET and children’s emotion expressions is bidirectional, such that children with dispositional tendencies to experience or express intense emotions might elicit certain types of parenting behaviors, including ET. In a longitudinal study of parental ET with toddlers in a predominantly Latino sample, Lorenzo et al. (2023) found that toddlers’ externalizing problems prospectively and positively predicted maternal ET, whereas maternal ET did not predict a child’s externalizing behaviors [18]. One interpretation is that the mothers whose children had high levels of externalizing behavior increased the use of ET to teach or scaffold their children’s emotion regulation in frustrating situations. However, Vu et al. (2022) found some support for bidirectional relations between mothers’ emotion socialization strategies (e.g., emotion dismissing and emotion coaching) and children’s (4–8 years of age) anger and sadness reactions to disappointment in Chinese immigrant families [19].

### 1.3. Cultural Variations in Parental ET and Children’s Emotion Expressions

Emotion models are mental representations of expected, valued, appropriate, and desirable emotional patterns within a cultural context, which serve to shape individual emotional experiences and expressions [20]. Although both Chinese and Mexican cultures are considered collective cultures, they differ in specific emotion values. In Chinese/Asian cultures, open expression of emotions in public is discouraged due to its potential threat to interpersonal harmony [21]. Moreover, the experience of low-arousal positive emotions (e.g., calm) is preferred over high-arousal positive emotions (e.g., excitement) in Chinese/Asian individuals [22]. Influenced by these cultural values, Chinese/Asian families might discourage children from experiencing and expressing high-arousal positive emotions, dampen children’s high-intensity positive emotion, or model emotion suppression [23]. In contrast, related to the Latino cultural values of *respeto* (i.e., obedience/conformity to authority, deference, decorum, and socially appropriate public behaviors [24], and *simpatía* (i.e., the preference for social interactions characterized by warmth and emotional positivity and avoidance of conflict/overt negativity [25], Latino cultures value open expressions of positive emotions while discouraging expressions of negative emotions. In line with these cultural values, compared to Latino-heritage (and European-heritage) young adults, Asian-heritage young adults rated positive emotions as the least desirable to experience and the least appropriate to express, whereas Latino-heritage young adults rated negative emotions as less desirable compared to Asian-heritage young adults [20]. Influenced by these cultural values, Latinx families might encourage and model open expressions of positive emotions and create opportunities to savor or enhance children’s positive emotions.

In line with theorized cultural influences on emotion socialization, past studies have found some evidence of cultural variations in parental ET. For example, Luo and colleagues (2014) [26] found that during parent–child shared book reading, Chinese mothers were most likely to emphasize negative consequences of inappropriate behaviors and referred to emotions less frequently than mothers from other U.S. ethnic groups (African American, Mexican, and Dominican families). In a previous study using data from the same sample as the present paper, we found that MA parents used more negative emotion words and emotion reasoning and engaged in more elaborate ET than did CA parents during shared picture book reading [9]. However, this study did not examine the link between parental ET and children’s emotion expressions.

Cultural variations in emotion values are also expected to shape children’s emotion expressions and emotion-related behaviors through emotion socialization. However, few studies have examined cultural group variations in children’s emotion expressions using observational methods, and the findings are mixed. For example, European American girls smiled more than Mainland Chinese and Chinese American girls and displayed higher disgust-related expressions and greater overall expressivity than Mainland Chinese girls [27]. Using two emotion situations (resistance to temptation and a “mishap” paradigm), Wang and Barrett (2015) [28] found that American preschoolers expressed more happiness and sadness than Chinese preschoolers. Although the two groups did not differ on overall expressiveness in anger, the Chinese children’s anger showed a cumulative pattern across contexts. The findings suggest that cultural group differences in emotional expressiveness may be “dimension-specific, emotion-specific, and context-specific” ([28] p. 420). On the other hand, Mexican American toddlers expressed more positive affect than negative affect during parent–child free play [29], consistent with Latino value of *simpatía* [30].

To our knowledge, few studies have sampled Mexican- and Chinese-heritage children in the same study and examined the similarities and differences in emotion expressions and emotion-related behaviors by cultural group. As an exception, using data from the same sample as the present study, we previously found that MA preschoolers displayed a higher intensity of positive (but not negative) emotions than CA preschoolers when treated unfairly (in an unfair sharing task), although the two groups did not differ in the expression of negative emotions [9]. Moreover, MA preschoolers displayed more frequent emotion-related behaviors than CA children, which may have been driven by the overall higher intensity or duration of emotion expressions [9]. However, the question remains whether the cultural group variations in children’s emotion expressions generalize to other situations (e.g., a frustration-eliciting task). Moreover, the study did not examine the links between parental ET and children’s emotion expressions and emotion-related behaviors.

When studying parental ET and children’s emotion expressions, it is important to consider other sociocultural and individual factors, such as gender/sex, parental education, and family income, which have been linked to emotion socialization. For example, a cumulative risk index capturing multiple family risks (e.g., low income, single-parent status, larger household size, and lower parental education) was associated with the mothers’ less frequent use of expressive encouragement and problem-focused responses in response to children’s negative emotions [31]. Moreover, the mothers’ education levels have been negatively related to their displays of unsupportive emotion reactions [31] and positively related to their use of positive emotion words, emotion questions, and emotion explanations [32]. Parents’ emotion socialization differed according to the child’s sex: parents of preschoolers engaged in more elaborate emotion conversations with their daughters than with their sons [33]. Moreover, parental education and child’s age were associated positively with parental emotion talk [9].

### 1.4. The Present Study

The present study has two aims. First, we used an observational approach to assess children’s emotion expressions (anger, sadness, and positive emotions) and emotion-related behaviors (attention to target, gaze aversion, and verbalization) during a frustration paradigm in two cultural groups: Chinese American and Mexican American preschoolers. We compared cultural group differences and similarities in the frequency of children’s emotion-related behaviors and intensity of emotion expressions. Based on prior findings [20,30,34], we hypothesized that MA children would express more positive affect than CA children. Due to past mixed findings on cultural differences in anger expression [28,34] and negative expressions [9], we tested cultural group differences in anger and sadness expression as an exploratory aim. This hypothesis will be tested using independent-sample t-tests. Because no prior studies have compared children from the two cultural groups on emotion-related behaviors, we tested this as an exploratory hypothesis.

Second, we examined the unique relations of parental ET to children’s emotion-related behaviors and emotion expression, controlling for other demographic factors (family SES, child’s age, sex, and generation status). This hypothesis will be tested using multiple regression analyses. Based on prior findings showing that parental ET is associated positively with child’s emotion regulation [16,32] and emotion understanding [35] in older children, we hypothesized that parental ET would be associated positively with children’s emotion-related behaviors. Due to lack of prior research on parental ET and children’s emotion expressions, we tested the relations between parental ET and children’s emotion expression as an exploratory hypothesis.

## 2. Materials and Methods

### 2.1. Participants

The study utilized data from a cross-sectional study on language and socioemotional development of dual language learners in immigrant families (data were collected between March 2013 and March 2016). The sample consisted of a total of 86 children (age range = 38 to 70 months, M = 54.4 months, SD = 7.11, 62% girls) and their parents, with 43 children from Mexican American (MA) families and 43 children from Chinese American (CA) families in a metropolitan area in Northern California. Three MA children and one CA child were excluded from the analysis because their video data were not captured due to equipment malfunctions during assessment. The average length of parental education was 9.6 years (equivalent to some high school, range = 2.5–16.0 years, SD = 3.84). Eighteen percent of children were first generation (born outside of the US), 78% were second generation (born in the U.S. with at least one foreign-born parent), and 6% were third generation (born in the U.S. with two U.S.-born parents). The average family per capita income in the past year was $5166.8 (range = $1000 to $24,166.7, SD = $5166.8).

We conducted independent sample t-tests (for continuous variables) and chi-square tests of independence (for categorical variables) to compare the MA and CA groups on demographic variables (child’s age, sex, generation status, parental education, and family income). The two groups differed on child’s generation status: the CA group had more first-generation children, and the MA group had more third-generation children, χ^2^(df = 2) = 23.4, *p* < 0.001. No group difference was found with parental education or family income. Furthermore, prior analyses conducted using data from the present sample found no significant cultural group differences in children’s English receptive and expressive vocabulary [9].

### 2.2. Procedures

Participants were recruited from 15 Head Start preschool centers in Northern California serving high concentrations of either Chinese-speaking or Spanish-speaking families. To be eligible, the child had to meet the following criteria: (1) be between 36–71 months of age, (2) be enrolled at a Head Start program for at least 3 days per week, (3) able understand and speak some English and either Chinese (Cantonese or Mandarin) or Spanish, (4) have both parents self-identifying as ethnically Chinese or Mexican. Children who were diagnosed with a speech or language disorder or who were receiving speech or language services were excluded from the study.

The parent–child dyad participated in a 2.5 h assessment, either at the university laboratory or at their homes, based on the parents’ preference. The assessment included parent questionnaires, one-on-one child emotion and behavioral tasks, and parent–child interaction tasks. Child participants were interviewed in their preferred language (18% in English and 82% in Cantonese/Mandarin or Spanish), during which they played games with bilingual research assistants and were tested on their linguistic and socioemotional development. Parents also completed parent questionnaires administered in their preferred language (10% in English, 90% in Cantonese/Mandarin or Spanish) by bilingual research assistants. Children were given a small prize, and parents received $70 for participation.

### 2.3. Measures

#### 2.3.1. Demographic Variables (Parent Report)

Parents were administered the adapted version of the Family and Demographics and Migration History Questionnaire [36] by a bilingual interviewer. Items included the child’s sex, the child’s age, the parent’s age, the parent’s length of stay in the US, maternal education, paternal education, annual per capita income, and child’s generation.

#### 2.3.2. Parental Emotion Talk (Observed)

Shared picture book reading task. We recorded parent–child book-sharing interactions using a picture book “Frog, where are you” [37] with no words. The book contains 29 black and white illustrations depicting a little boy’s journey to find his lost frog. Parents were given 5 min to read the story with their child in their preferred language, which could be English, Cantonese, Mandarin, or Spanish. The visual images in the book evoke various emotions, such as happiness, anger, fear, and sadness, prompting parents and children to engage in emotion talk during the storytelling process. Shared book reading tasks are commonly used in research on parent–child interactions as they can elicit rich and complex language from parents, including emotion talk [17]. For example, Huang and Kan (2021) [38] used three picture book storytelling tasks to examine parental emotion talk in Cantonese-speaking Chinese American immigrant families with preschool-age children and found that Chinese American parents’ use of emotion words and emotion reasoning did not differ across the books.

Emotion talk coding scheme. The Frog Storytelling Task recordings were transcribed and scored using a coding manual adapted from Spinrad and Eisenberg (2010) [39], which had been previously validated in a sample of Chinese American families with school-age children [6,32]. Two bilingual coders proficient in both Chinese and English coded the Chinese American sample, whereas two bilingual coders proficient in Spanish and English coded the Mexican American sample. The coders worked independently on each sample and resolved discrepancies through post-discussions. The measurement of emotion talk (ET) included three components: the number of emotion words, the number of ET comments and questions, and the overall elaborateness of ET. Interrater reliabilities, assessed using intra-class correlations (ICCs), were 0.93 for parental emotion words, 0.71 for parental emotion questions, 0.74 for parental emotion reasoning, and 0.62 for overall ET elaborateness. According to Koo and Li (2016) [40], ICCs between 0.50 and 0.75 are considered to indicate moderate reliability, and those between 0.75 and 0.90 are considered to indicate good reliability. Therefore, the ET codes demonstrated moderate-to-good interrater reliabilities in the present sample. Only parental ET variables were used in the analysis because child ET variables had low frequencies and little variability in the present sample.

Emotion words. The coders counted the number of emotion words uttered by the parent during the book-reading task. Emotion words included words that express affective states (e.g., “mad” 生氣/enojada, “unhappy” 不開心/infeliz, “afraid” 害怕/temerosa, …) and words that are related to emotional states without naming the specific emotion (e.g., “laugh”, “naughty”, “tired” …) The counted emotion words were categorized as having a positive or negative valance.

Emotion talk comments and questions. Three types of parental emotion comments or questions were categorized, and the frequencies of utterance were counted: linking, emotion questions, and emotion reasoning. Linking refers to parental linking of the story to personal experiences (e.g., “I told you; bees sting you because they get mad since the beehive is their home”). Emotion questions refer to a parent’s raising of questions regarding the character or the child’s emotional state (e.g., “Why is the boy mad?”). Emotion reasoning refers to a parent providing explanations for a character’s feeling (e.g., “The little boy is happy because he saw that the little frog is with its family”).

Global quality (elaborateness) of emotion talk. The coders rated each parent on a global code of ET quality based on the parent’s storytelling behaviors throughout the whole task, using a 3-point scale (1 = no ET present, 2 = low quality of ET, 3 = high quality of ET). Coders evaluated the quality of ET based on the elaborateness of ET (the levels of detail and sophistication, the amount of information conveyed regarding emotions, and the degree to which parents engaged children in the discussion of emotions). Low quality of ET (a rating of 2) was represented by use of short statements and simple questions, such as “The boy is sad” and “Is the boy unhappy?” High quality of ET (a rating of 3) was represented by the use of detailed explanations of emotions (e.g., “The boy got angry because the jar is broken, right?”), questions that elicits the child’s own thoughts (e.g., “What do you think he is feeling?”), and sentences that take the perspective of the character (e.g., “The boy named Luis scolded the dog and told him that he needed to behave”).

Data reduction of ET variables. To reduce the number of parental ET variables, we conducted an exploratory factor analysis with the seven ET variables using Principal Axis Factoring. Based on the scree plot, one factor was extracted, which accounted for 59% of total variance. Two ET variables (linking and self-reporting of emotion) did not load onto the factor and were dropped from subsequent analyses. The other five ET variables had factor loadings larger than 0.30: positive emotion words (0.33), emotion questions (0.47), overall quality of ET (0.67), emotional reasoning (0.74), and negative emotion words (0.80). Based on these results, we computed a composite score of parental ET by averaging the standardized scores of the above five ET variables, with a higher score on the ET composite indicating more frequent ET behaviors and more elaborate ET.

#### 2.3.3. Children’s Emotion Expressions and Emotion-Related Behaviors (Observed)

Attractive toy in a transparent box task. The LAB–TAB Preschool Battery’s Attractive Toy in a Transparent Box Task [41] was utilized to evaluate children’s emotion expression and regulation. During this task, children were asked to select a toy they liked, which was then locked in a transparent box by the experimenter. The child was given a set of incorrect keys and instructed to use them to open the box and access the toy. After a 4 min interval, the experimenter returned, apologized, and provided the child with the correct key, allowing them to finally play with the toy.

Coding scheme. The task was recorded on video and analyzed using a coding scheme that included codes for affect and behavior. Emotion expression was measured by the intensity and duration of the observed affect, and emotion-related behaviors were measured by the frequency of observed behaviors. The videos, which were approximately 6–7 min in length, were segmented into 5 s epochs using ELAN (Version 5.9), a psycholinguistic software [42]. An average of 74.8 epochs and 77.5 epochs were coded for each video in the MA and CA samples, respectively. Two trained coders who were proficient in Spanish coded the MA sample, while two trained coders who were proficient in Cantonese and Mandarin coded the CA sample. The coders scored the videos independently and resolved any differences in coding through consensus meetings.

Emotion expression codes. The coders assessed children’s expression of anger, sadness, and positive affect in each 5 s epoch, taking into consideration facial, vocal, and bodily displays. Emotion expression was rated on a 4-point scale, where 1 indicated the absence of emotion, 2 indicated low-intensity expression, 3 denoted moderate-intensity expression or prolonged low-intensity expression, and 4 indicated intense expression or prolonged moderate-intensity expression. For each emotion expression, the ratings were averaged across all epochs. Interrater reliabilities (computed as intraclass correlations/ICCs) between the two coders were 0.93 for expressed positive affect, 0.82 for expressed anger, and 0.52 for expressed sadness.

Emotion-related behavioral codes. Twelve behavioral codes were employed to capture children’s emotion-related behaviors during the task. In the current study, four of these behavioral variables were selected and coded in 5 s epochs, namely attention to target objects, gaze aversion, feeling–state language, and non-feeling–state language. Codes of 0 were used to indicate the absence of a behavior, whereas codes of 1 represented the presence of a behavior. The average of codes across all epochs was used for each emotion-related behavior variable in the data analyses. ICCs between coders were acceptable: 0.92 for attention to target, 0.88 for gaze aversion, 0.69 for feeling–state language, and 0.97 for non-feeling–state language. The feeling–state language variable was dropped due to extremely low frequency.

### 2.4. Data Analytic Plan

First, after data screening and descriptive analyses, independent-sample t-tests were performed to test for cultural group differences in the means of parental ET and children’s emotion variables. Second, zero-order correlations were computed to examine the associations between socio-demographic variables and parental ET and children’s emotion expressions. Socio-demographic variables that were significantly correlated with both parental ET and children’s emotion expressions were included as covariates in subsequent analyses testing the relations between parental ET and child’s emotion variables. Third, multiple regressions were computed to test the unique relations between parental ET and children’s emotion expressions/behaviors, controlling for socio-demographic covariates. All analyses were conducted in IBM SPSS (Version 27).

## 3. Results

### 3.1. Descriptive Statistics and Cultural Group Comparisons

Descriptive statistics for parental emotion talk (ET), children’s emotion expression variables, and demographic variables are reported in Table 1. Based on the cutoffs of 2 and 7 for skewness and kurtosis respectively [43], all the study variables were normally distributed. There were significant differences between the MA and CA groups: (a) in parental ET, MA parents scored higher, t(df = 78) = 3.75, *p* < 0.001, Cohen’s d = 0.65; (b) in children’s expressed anger, MA children displayed more anger, t(df = 84) = 9.99, *p* < 0.001, Cohen’s d = 0.26; and (c) in children’s expression of sadness, MA children scored higher, t(df = 84) = 3.01, *p* = 0.003, Cohen’s d = 0.23.

### 3.2. Correlations Among Demographic and Study Variables

Zero-order correlations among parental ET and children’s emotion expression and emotion-related behaviors, as well as demographic variables (child’s sex, age, generation status, and family SES), are presented in Table 2. Significant positive correlations were found between the quality and elaborateness of parental ET and children’s expressions of anger (r = 0.32) and sadness (r = 0.28), and use of non-feeling state language (r = 0.27) during the frustration task. It is worth noting that children’s emotion expression variables were mostly unrelated to each other, except for a positive correlation between expressed anger and expressed sadness (r = 0.32) and a negative correlation between gaze aversion and attention to target (r = −0.96).

Regarding the demographic variables, child’s age was positively correlated with children’s expressions of anger (r = 0.27) and use of non-feeling state language (r = 0.23). Children’s generational status was positively correlated with their use of anger, such that second- or third-generation children expressed more anger than first-generation children (r = 0.32). Child’s sex or family’s SES was unrelated to any parental ET or child’s emotion expression or regulation variables. Based on the correlation results, we included child’s age and generation status as covariates in subsequent analyses, testing the relations between parental ET and child emotion variables.

### 3.3. Multiple Regressions Testing the Relations of Parental ET to Children’s Emotion Expressions

Six multiple regressions were computed to test the relation of the parental ET composite to each of the six children’s emotion expression variables, controlling for child’s age and generation status. As shown in Table 3, parental ET, child’s age, and generation status were entered as simultaneous predictors. The regression equation accounted for significance variances in three dependent variables: child’s expressed anger (R^2^ = 0.23, *p* < 0.001), expressed sadness (R^2^ = 0.12, *p* < 0.05), and non-feeling state language (R^2^ = 0.21, *p* < 0.001). Specifically, child’s generation status (ß = 0.28) and parental ET (ß = 0.28) were positively associated with child’s expressed anger. Parental ET was positively associated with child’s expressed sadness (ß = 0.25). Moreover, child’s age (ß = 0.38) and parental ET (ß = 0.27) were positively associated with child’s use of non-feeling state language. For the six regression models tested in Table 3, we further tested whether culture group moderated the relation between parental ET and child outcomes by adding the culture group (0 = MA, 1 = CA) and the multiplicative term of culture group × parental ET as predictors. None of the interaction terms was significant. Thus, no evidence of moderation by cultural group was found.

## 4. Discussion

This is one of the first observational studies that examined the link between parental ET and children’s emotion expressions and emotion-related behaviors among low-income Mexican American and Chinese American families. Using independent-sample t-tests, we found that MA children expressed more anger and sadness during the frustration task compared to CA children, although the two groups did not differ in expressed positive emotions or emotion-related behaviors. Moreover, MA parents used more ET during shared book reading than did CA parents. Using multiple regressions and controlling for socio-demographic covariates, we found that the children whose parents used more ET in shared reading expressed more anger and sadness and used more non-feeling states languages during the frustration paradigm.

Tested as an exploratory hypothesis, we found that MA children displayed more anger and sadness during the frustration task than CA children. Because no prior studies have directly compared MA and CA children from similar socioeconomic background on emotion expressions using observational measures, we can only compare the study results with the finding of Kim et al. (2023) [9], which used observational data from the same sample but with a different emotion paradigm (unfair treatment). Contrary to our results, Kim et al. (2023) [9] did not find cultural group differences in children’s expressions of anger during the unfair sharing paradigm. It is important to note that the frustration task (attractive toy in a transparent box) examined in the present paper differs from the unfair sharing task examined in Kim et al. (2023) [9]: the unfair sharing task involves social interactions with an adult (the interviewer shares a bag of candies with the child), whereas the frustration task involves minimum interactions with the social partner (the child was asked to open the transparent box on their own without the interviewer present). Thus, the two situations differ in the emotion display rules. Although it might seem impolite or inappropriate for children to express negative emotions when interacting with an adult in both Latino and East Asian cultures, displaying negative emotions while trying to solve a frustrating problem on one’s own is not necessarily viewed as inappropriate in either Latino or East Asian cultures. Therefore, culturally based emotion models are situation specific. While there are general cultural variations in the desirability and appropriateness of expressing different types of emotions, the emotion display rules depend on the interpersonal and personal goals of specific situations. It is interesting that preschool-age children in U.S. immigrant families living in the same geographic region are displaying divergent patterns of situation-specific emotion expressions that are consistent with the emotion models of their heritage cultures.

On the other hand, contrary to our hypothesis that MA children would display more positive emotions than CA children, we did not find significant culture group difference in positive emotion expressions. This is different from Kim et al. (2023) [9], which found that MA children displayed more positive emotions and emotion-related behaviors (gaze aversion, self-soothing, and fidgeting) than CA children during the unfair sharing paradigm. However, an examination of group means (in Table 1) suggested that the result is in the expected direction: MA children (M = 1.55) had a higher mean in expressed positive emotions than CA children (M = 1.44) in the frustration paradigm, although it did not reach statistical significance. Perhaps the frustration paradigm offered limited opportunity for observing children’s positive emotions. Therefore, the situational context of the frustration paradigm, coupled with the small sample size, might have limited our ability to detect cultural group differences in expressed positive emotions.

Our second exploratory aim was to examine the unique relations between parental emotion talk and children’s emotion expression and emotion-related behaviors. Using a series of multiple regressions, we found that after controlling for child’s age and generation status, parental ET positively predicted children’s expressed anger and sadness and their use of non-feeling state languages. Thus, the children whose parents used more ET during shared book reading expressed more anger and sadness and used more non-feeling state language during the frustration task than the children whose parents used less emotion talk. These results are generally in line with prior studies showing that parental ET was associated with child’s emotion regulation [16,32] and emotion understanding [35], although no prior studies have examined the specific links between ET and preschool-age children’s emotion expressions. Overall, our results indicate a general pattern that parents who used more ET tended to have children who are more emotionally expressive. Although we did not examine the mechanisms that might underly this association, we can speculate on a few possibilities. First, parents who use more ET might convey to children that it is okay to express and talk about emotions (via shaping children’s emotion control values), which in turn can shape children’s emotion expressions and emotion-related behaviors [14,15]. Second, it is also possible that children who are temperamentally more expressive elicit more ET in parents (e.g., by creating more opportunities for parents to respond to and talk about children’s emotions or by making parents more attuned to children’s emotions and more motivated to provide scaffolding for children’s emotion regulation [18]. Future studies should replicate these relations in other samples and investigate the mechanisms using longitudinal data.

## 5. Limitations and Conclusions

The study has several limitations. First, the cross-sectional design did not allow us to test the directionality of the relations between parental ET and child’s emotions, which is of theoretical importance given the transactional nature of parent–child interactions. Second, the small sample size offered limited statistical power for testing cultural group variations in strengths of relations between ET and emotion expressions. Third, the interrater reliability for children’s expressed sadness was low, partly because children were allowed to move freely during the tasks, and their facial expressions were often obstructed by the box or shadow, making it difficult to code expressed sadness (which involves more subtle facial expressions and behaviors than anger or positive emotions). Also, we did not code other types of negative emotions (e.g., fear, anxiety). Fourth, the brief frustration task did not provide sufficient opportunity to observe a full range of emotion-related behaviors during this age period, such as self-soothing, self-talk, and problem-solving behaviors. Future research should use multiple methods and tools to assess children’s emotion expressions and emotion-related behaviors across different situations to obtain a better understanding of cultural variations in emotion models. Fifth, the shared picture book reading task might not capture the full range of parent–child ET in daily life. Moreover, because the ET coding theme was originally developed in English based on conversations in European American families and only includes explicit verbal references to emotions, it might not capture subtle or indirect references to emotions in Spanish or Chinese languages. Future research can use qualitative or mixed methods to capture unique aspects of parent–child discourse related to emotions in Mexican and Chinese families.

In sum, this study revealed culture group similarities and differences in preschool-age children’s emotion expressions and emotion-related behaviors in low-income immigrant families. Regardless of culture, children whose parents used more emotion talk during shared book reading expressed more anger and sadness and used more non-feeling states languages in the frustration-eliciting situation. The findings highlighted emotion talk as a potential mechanism through which culturally based values and norms in emotion expressions are transmitted. The findings have implications for school-based socioemotional learning and parent psycho-education programs for promoting socioemotional development in early childhood. Specifically, these programs need to be sensitive to cultural variations in how parents and children express and communicate emotionally, and cultural adaptations might be necessary to enhance the programs’ fit with and potential to engage immigrant families from diverse backgrounds.

## Figures and Tables

**Table 1:** Descriptive statistics of study variables by cultural groups.

Variables	Mexican American Sample (*n* = 43)		Chinese American Sample (*n* = 43)	
	Mean	SD	Mean	SD
Parental emotion talk ^1^	0.26 _a_	0.65	−0.29 _b_	0.65
Child’s positive emotions ^2^	1.55	0.31	1.44	0.38
Child’s anger ^2^	1.70 _a_	0.34	1.16 _b_	0.11
Child’s sadness ^2^	1.37 _a_	0.25	1.22 _b_	0.22
Child’s attention to target ^3^	0.85	0.10	0.85	0.13
Child’s gaze aversion ^3^	0.17	0.11	0.19	0.15
Child’s non-feeling state language ^3^	0.12	0.11	0.08	0.12
Child’s age	55.22	6.96	53.48	7.23
Average parent education	10.28	3.29	8.90	4.26
Per capita income	$4664	$3875	$5646.9	$3406.8
Family SES composite ^4^	0.04	0.65	−0.04	0.77

Notes. ^1^ Parental emotion talk is a composite computed as the mean of standardized scores of the five ET variables (positive and negative emotion words, emotion questions and reasoning, and overall elaborateness of ET). ^2^ Child’s positive emotions, anger, and sadness are the mean ratings across epochs coded on a 4-point scale (from 1 = absence of emotion expression to 4 = intense expression or prolonged moderate intensity expression). ^3^ Child’s attention to target, gaze aversion, and non-feeling state language are computed as the average presence (0 = not present, 1 = present) of such behavior across all epochs. ^4^ Family SES composite was computed as the mean of standardized parental education and family income variables. Means with different subscripts differ at *p* = 0.05 level across the two cultural groups (MA vs. CA) by independent-sample *t*-tests.

**Table 2:** Zero-order correlations among demographic and study variables.

Variables	1	2	3	4	5	6	7	8	9	10
1. Child’s sex ^1^	–									
2. Child’s age	0.02	–								
3. Child’s generation status ^2^	−0.02	0.19	–							
4. Family’s SES	−0.09	0.07	0.14	–						
5. Parental emotion talk	−0.05	0.03	0.12	0.16	–					
6. Child’s expressed positive emotions	−0.07	0.12	0.13	−0.05	−0.07	–				
7. Child’s expressed anger	−0.14	0.27 **	0.33 **	0.12	0.32 **	0.04	–			
8. Child’s expressed sadness	−0.05	0.04	0.19	0.03	0.28 *	−0.10	0.32 **	–		
9. Child’s attention to target	0.07	0.20	−0.09	0.16	0.06	−0.04	0.17	−0.10	–	
10. Child’s gaze aversion	−0.13	−0.18	0.04	−0.15	−0.12	0.02	−0.21	0.03	−0.95 ***	–
11. Child’s non-feeling state language	−0.12	0.23 *	0.03	−0.10	0.27 *	0.14	0.10	0.07	0.03	−0.03

Notes. ^1^ Child’s sex is coded as follows: 0 = girls, 1 = boys; ^2^ Child’s generation is coded as: 1 = 1st generation, 2 = 2nd generation, 3 = 3rd generation. *** *p* < 0.001, ** *p* < 0.01, * *p* < 0.05.

**Table 3:** Multiple regressions predicting children’s emotion expressions from parental emotion talk.

**Predictors**	**Regression 1: Predicting Child’s Expressed Positive Affect**		**Regression 2: Predicting Child’s Expressed Anger**		**Regression 3: Predicting Child’s Expressed Sadness**	
	**ß**	**B (SE)**	**ß**	**B (SE)**	**ß**	**B (SE)**
Child’s age	0.13	0.01 (0.01)	0.18	0.01 (0.01)	−0.07	−0.02 (0.004)
Child’s generation status	0.13	0.09 (0.09)	0.28	0.22 * (0.08)	0.21	0.10 (0.05)
Parental emotion talk	−0.09	−0.05 (0.06)	0.28	0.15 ** (0.05)	0.25	0.08 * (0.04)
	R^2^ = 0.05		R^2^ = 0.23 ***		R^2^ = 0.12 *	
**Predictors**	**Regression 4: Predicting Child’s Attention to Target**		**Regression 5: Predicting Child’s Gaze Aversion**		**Regression 6: Predicting Child’s Non-feeling State Language**	
	**ß**	**B (SE)**	**ß**	**B (SE)**	**ß**	**B (SE)**
Child’s age	0.20	0.003 (0.002)	−0.16	−0.003 (0.002)	0.38	0.01 *** (0.002)
Child’s generation status	−0.10	−0.02 (0.03)	0.05	0.12 (0.03)	−0.10	−0.02 (0.02)
Parental emotion talk	0.07	0.01 (0.02)	−0.12	−0.02 (0.02)	0.27	0.04 * (0.02)
	R^2^ = 0.04		R^2^ = 0.04		R^2^ = 0.21 ***	

Notes. ß = standardized regression coefficient; B = unstandardized regression coefficient; SE = standard error of regression coefficient. * *p* < 0.05, ** *p* < 0.01, *** *p* < 0.001.

## Data Availability

The data is available upon request due to privacy and ethical reasons.
